# Deep Residual CNN-Based Ocular Recognition Based on Rough Pupil Detection in the Images by NIR Camera Sensor

**DOI:** 10.3390/s19040842

**Published:** 2019-02-18

**Authors:** Young Won Lee, Ki Wan Kim, Toan Minh Hoang, Muhammad Arsalan, Kang Ryoung Park

**Affiliations:** Division of Electronics and Electrical Engineering, Dongguk University, 30 Pildong-ro 1-gil, Jung-gu, Seoul 04620, Korea; lyw941021@dongguk.edu (Y.W.L.); yawara18@dongguk.edu (K.W.K.); hoangminhtoant2l1@gmail.com (T.M.H.); arsal@dongguk.edu (M.A.)

**Keywords:** biometrics, rough pupil detection, ocular recognition, deep ResNet

## Abstract

Accurate segmentation of the iris area in input images has a significant effect on the accuracy of iris recognition and is a very important preprocessing step in the overall iris recognition process. In previous studies on iris recognition, however, the accuracy of iris segmentation was reduced when the images of captured irises were of low quality due to problems such as optical and motion blurring, thick eyelashes, and light reflected from eyeglasses. Deep learning-based iris segmentation has been proposed to improve accuracy, but its disadvantage is that it requires a long processing time. To resolve this problem, this study proposes a new method that quickly finds a rough iris box area without accurately segmenting the iris region in the input images and performs ocular recognition based on this. To address this problem of reduced accuracy, the recognition is performed using the ocular area, which is a little larger than the iris area, and a deep residual network (ResNet) is used to resolve the problem of reduced recognition rates due to misalignment between the enrolled and recognition iris images. Experiments were performed using three databases: Institute of Automation Chinese Academy of Sciences (CASIA)-Iris-Distance, CASIA-Iris-Lamp, and CASIA-Iris-Thousand. They confirmed that the method proposed in this study had a higher recognition accuracy than existing methods.

## 1. Introduction

Due to recent developments in technology, iris recognition is being used for personal authentication in smartphones [1]. The basic concept of iris recognition that is used conventionally was first proposed in a patent by Flom et al. [2], and the fundamental basis for most iris recognition systems that are currently used was created by Daugman et al. [3,4,5,6]. Generally, iris recognition performance is influenced by the environment in which the image is captured (e.g., a noisy environment, low resolution, optical or motion blurring, specular reflection and in-plane rotation, off-angle, off-axis). To prevent such environmental influences, iris images were captured in a constrained environment. Furthermore, there were cases in which iris recognition with a visible camera was difficult in Asian people due to the dark color of their irises, which is caused by a high level of the pigment melanin. If the images were captured by a near-infrared (NIR) light camera sensor, full iris features could be obtained despite a dark iris color [7,8,9,10,11,12]. However, an NIR camera requires an NIR illuminator, and using the latter increases the size and price of the system. Therefore, studies are still being conducted on iris recognition using images captured by visible camera sensors, which are relatively easy to set up [13,14,15,16]. However, there are cases in which more noise can occur when a visible camera is used, resulting in a reduced recognition performance. Problems such as optical or motion blur, specular reflection, and in-plane rotation can occur when iris images are captured with both NIR and visible light cameras. If this occurs, it becomes difficult to accurately segment the iris area, which could have a significant effect on the accuracy of iris recognition. Segmenting the iris area is also a very important preprocessing step in the overall iris recognition process. The problems that occur in a constrained image acquisition environment become even more severe in an unconstrained environment. To resolve these problems, more acquisition system designed in a more complex manner are required, along with high quality images and processing performances [7,8,13,14]. 

There are studies which resolve those problems by using an ocular area that is wider than the conventional iris area [17]. The ocular area can be used in applications in which a confirmation of identity is performed through the surrounding skin when an image of the iris cannot be captured reliably. Furthermore, if the image is captured from a long distance, its resolution is low and the recognition performance is reduced because of this. Using the ocular area has the advantage of improving performance in such cases; however, it is still difficult to extract the features that are important for personal recognition from ocular areas outside the iris, including skin with little texture. In addition, there are still problems with reduced recognition rates due to misalignment between the enrolled and recognition ocular images. To consider these issues, we propose deep learning-based ocular recognition based on rough pupil detection.

This paper is organized as follows: Section 2 compares the advantages and disadvantages of existing iris and ocular recognition methods. Section 3 describes the contributions of this study and Section 4 shows ocular recognition using the deep residual network (ResNet) proposed in this study. Section 5 presents the experimental results and analysis, and Section 6 presents the conclusions of the study.

## 2. Related Works

In iris recognition, the iris segmentation performance and texture quality in the captured image are greatly affected by the image capturing environment. Thus, this is one aspect that makes it difficult to create an iris recognition system for a variety of environments. To resolve this, the development of recognition methods that use the ocular region, which is somewhat larger than the iris region, has been attempted [17,18,19,20,21,22,23,24,25]. Rattani et al. [19] introduced a variety of studies on existing ocular recognition methods. In order to extract features from a visible input image using the ocular region, Park et al. [17] used a Hough transformation to detect the iris area and performed interest point detection and region sampling based on the detected image. After image alignment, global descriptors and features were extracted, and then a scale invariant feature transform (SIFT) was performed using gradient orientation (GO) and local binary pattern (LBP) as local descriptors in order to extract local features and perform matching [17]. Cho et al. [20] proposed a method that can improve recognition accuracy through adaptive bit shifting and matching after defining circular regions of interest (ROI) for creating a periocular area based on the iris radius and then converting this area to polar coordinates to make it robust against eye rotation. Rodriguez et al. [21] studied ocular recognition using a quadratic correlation filter. Ross et al. [18] studied ocular recognition through matching score fusion using various feature extraction techniques, such as gradient orientation histograms (GOH), modified SIFT (m-SIFT), and probabilistic deformation models (PDM).

In addition to this, they studied methods that can perform fusion and recognition to achieve the advantages of both iris and ocular recognition [22,23,24,25,26]. Boddeti et al. [22] studied matching score-level fusion of the iris and ocular regions through fusion optimal trade-off synthetic discriminant function (OTSDF) correlation filtering, and maximum a posterior probability (MAP) estimation in order to perform recognition on challenging ocular images captured in an NIR camera sensor environment. Verma et al. [23] performed periocular recognition on ocular images captured in a long-distance NIR camera sensor environment using traditional iris recognition, a pyramid histogram of oriented gradients (PHOG), and gist methods and then performed fusion and classification using a random decision forest (RDF). Oishi et al. [24] performed a study on the fusion of both iris and periocular recognition in a mobile device using adaptive boosting (Adaboost). Tan et al. [25] proposed a method of score fusion-based iris and periocular recognition using images captured by a visible light camera sensor in an uncooperative environment. Lee et al. [26] proposed a recognition method that performs a fusion on the results by inputting three different areas including the existing segmented iris area and a wider area around it into three convolutional neural network (CNN) models. Moreover, Ahuja et al. [27] proposed a recognition method that fused the recognition results of the iris area, the ocular area, and an area slightly larger than the ocular area. Processing of the ocular region was performed using features learned through the VisobNet CNN. Processing of the iris region was performed using features extracted through prior segmentation processing and root-SIFT. Processing of the face was performed using the OpenFace face recognition method [28]. However, the methods in these existing studies have issues in that recognition performance is affected by ocular region detection accuracy and the recognition accuracy is worse than iris segmentation-based iris recognition.

In previous research [29], a super-resolution reconstruction method based on CNN for iris recognition was proposed. In [30], the authors proposed periocular recognition by explicit attention to critical regions in deep CNN. Drozdowski et al. proposed the method of detection of glasses in ocular images [31]. In previous research [32], a classification method of heterogeneous objects including ocular images was proposed. Reddy et al. proposed the multi-frame super resolution for ocular recognition [33]. Although it is not ocular recognition but face recognition, Ahonen et al. proposed LBP-based face recognition with its application, and their method showed the promising recognition accuracies [34]. 

To address the problems of previous researches, this study proposes an ocular recognition method that quickly selects a rough iris box area without accurate iris segmentation. To address the problem of the reduced accuracy of recognition based on the rough selection, an ocular area somewhat larger than the iris area is used to perform recognition, and a deep ResNet is used to resolve the problem of a reduced recognition rate caused by misalignment between the enrolled and recognition images.

Table 1 is a summarized comparison of the method proposed in this study and existing methods. In Table 1, “A”–“N” means “CASIA-Iris-Distance database”, “NICE.II training database”, “face and ocular challenge series (FOCS) database”, “face recognition grand challenge (FRGC) database”, “mobile iris challenge evaluation (MICHE) database”, “CASIA-Iris-Lamp database”, “CASIA-Iris-Thousand database”, “self-collected dataset database”, “Q-FIRE iris dataset”, “CASIA cross sensor iris dataset”, “ND-iris-0405”, “ND-CrossSensor-Iris-2013”, “MBGC NIR v2 database”, and “VISOB dataset”, respectively.

## 3. Contributions

Our research is novel in the following four ways compared to previous works:

- We propose a NIR camera-based ocular recognition method using deep features without iris segmentation, as shown in Table 1.
- It uses a roughly selected eye area for recognition, without executing a specific process for detecting the pupil and iris boundary, the eyelid, and the eyelashes, as this process has a long processing time and its performance is affected by the image quality. To prevent a decrease in recognition performance due to the roughly selected eye area, an ocular area that is somewhat larger than the iris area is used to perform recognition. In addition, it uses a deep ResNet to resolve the problem of misalignment between the enrolled and recognition images that can occur due to recognition based on a roughly detected eye area, as well as the problem of reduced recognition performance caused by this misalignment.
- We analyze the feature maps from various convolutional layers of the deep ResNet in order to examine the features that are important for ocular recognition in each of the layers.
- We make our algorithm for rough pupil detection and ResNet models trained with three open databases (Dongguk CNN Model for NIR Ocular Recognition (DC4NO)) available to other researchers for the fair comparisons as shown in [39].

## 4. Proposed Method

### 4.1. Overall Procedure of Proposed Ocular Recognition Method 

**Figure 1:**
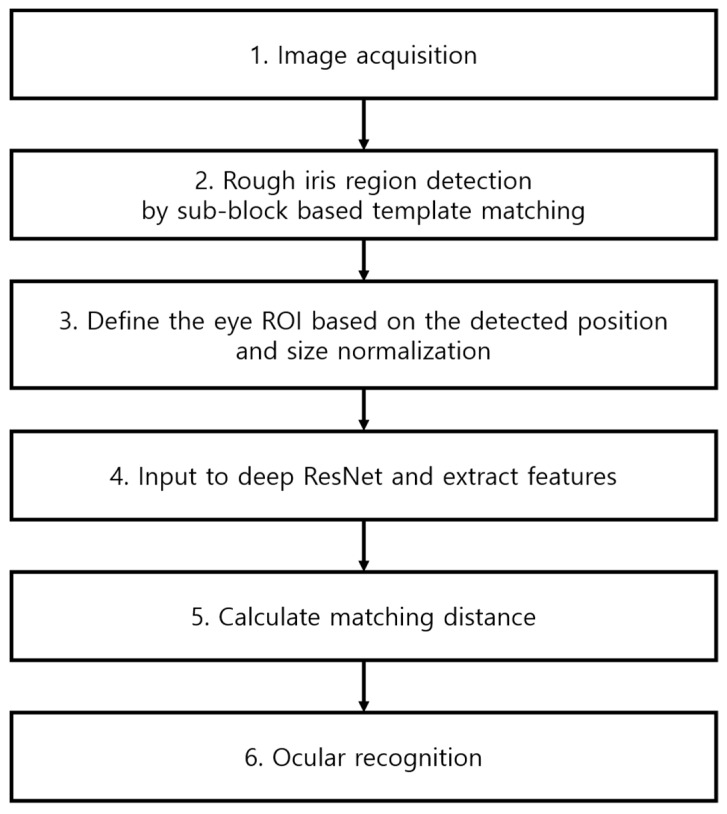
Overview of the proposed system.

Figure 1 shows an overall flowchart of the algorithm proposed in this study. A sub-block based template matching method is used to find a rough pupil area from the input iris image captured by the NIR camera sensor (Step 2 of Figure 1).

A somewhat larger ocular ROI is specified based on the selected rough pupil location, and a size normalization to 224 × 224 pixels is performed on this ROI to use it as input in the deep ResNet that is used in this study (Step 3 of Figure 1). In the next step, the feature vectors of the extracted input ROI are extracted from the deep ResNet (Step 4 of Figure 1). The matching distance to the pre-enrolled feature vectors is determined (Step 5 of Figure 1). If this distance is below a defined threshold, the input image is recognized as being in the same class as the pre-enrolled image (acceptance as genuine class). If the distance is greater than the threshold, the input image is rejected and considered as being in a different class from the pre-enrolled image (rejection as imposter class) (Step 6 of Figure 1). Section 4.2, Section 4.3 and Section 4.4 provide a more detailed description of this.

### 4.2. Rough Pupil Detection and Defining Ocular ROI

**Figure 2:**
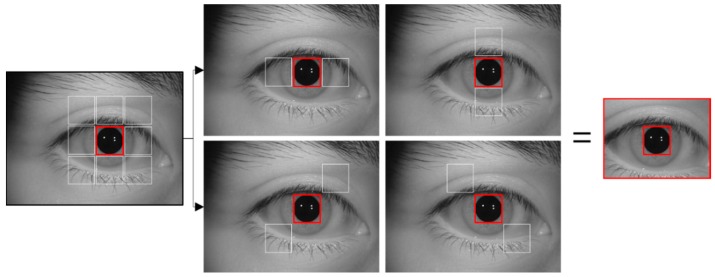
Example of rough iris area detection using sub-block-based template matching.

This study performs sub-block-based template matching to find a rough iris area in the input iris images. This algorithm uses a sub-block based template to find a rough eye area. The central sub-block is set as the initial search location, and eight sub-block areas are set in the up, down, left, right, and diagonal directions. After this, the mean values of the pixels with the areas in the up, down, left, right, and diagonal directions are compared. If the center value is not the lowest value, the algorithm moves to the next search area, and the process is repeated to find the location with the lowest value [40]. Moreover, to quickly search an image with this process, an integral image is calculated, and processing is performed based on this [41]. The sum of the area’s pixels obtained in this way is calculated for nine sub-blocks, as shown in Figure 2. Then, the position where the mean pixel value of the central sub-block in a certain location and the mean pixel value of its surrounding eight sub-blocks become the minimum vale is determined as the rough pupil location.

Although we used the sub-block-based template matching using 3 × 3 sub-blocks whose concept is similar to that in [40], there is novel enhancement in our research as follows. In [40], they calculate the difference between the mean of central sub-block and those of surrounding eight sub-blocks at the same time, which increases the processing time. However, in our method, the difference between the mean of central sub-block and those of surrounding two sub-blocks is checked sequentially. In details, the difference between that of central sub-block and those of two horizontal sub-blocks is checked as the first step, and the pupil candidate regions where this difference is small are predicted. Then, with these candidates, the difference between that of central sub-block and those of two diagonal sub-blocks is checked as the second step, and this procedure is repeated for the remained surrounding sub-blocks as shown in Figure 2. Based on this sequential and hierarchical step, the processing speed of our sub-block-based template matching is much enhanced compared to [40]. 

To consider the cases in which the size of the pupil area varies within the input image due to differences in the z-distance from the camera to the user’s eye, the sub-block’s size is altered, and the search is adaptively performed. In detail, the initial size of sub-block is 30 × 30 pixels with the adaptation to maximum 100 × 100 pixels. The stride is constant as 1 pixel in all the cases of sub-block-based template matching. The minimum value of sub-block template matching is calculated by comparing the previously saved minimum value (the difference between the mean of central sub-block and those of surrounding two sub-blocks at previous position) with the value calculated at current position. For example, if the value at the current position is smaller than that at previous position, the minimum value is updated by that at the current position whereas it is not updated if not as shown in Equation (1):

*DV_min_* = *DV_cur_* (if *DV_cur_* < *DV_prev_*) = *DV_prev_* (else if *DV_cur_* ≥ *DV_prev_*) (1)

where *DV_cur_* and *DV_prev_* are the difference values between the mean of central sub-block and those of surrounding two sub-blocks at current and previous positions, respectively. *DV_min_* is the minimum difference value. The step of increasing sub-block size is constant as 10 pixel in all the cases of sub-block-based template matching. If the condition is fulfilled for many sizes of sub-blocks, only one sub-block size is determined, which shows the minimum difference value of Equation (1) although it is sub-optimal case. That is because we do not intend to detect accurate ROI for recognition, and the detected ROI is not ideal and has a little positional errors. However, these could be compensated by our deep CNN. 

Afterward, an ocular ROI for the CNN input is defined based on the found pupil area, and this ROI is resized via bilinear interpolation. This kind of strategy of rough pupil detection and defining ocular ROI for deep CNN belongs to our proposed method.

**Figure 3:**
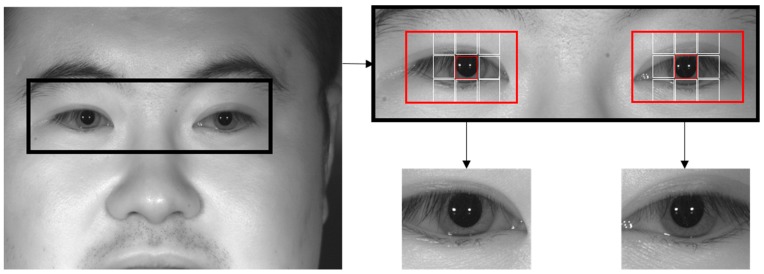
Example of rough detection of ocular ROI in CASIA-Iris-Distance database.

Of the three open databases used in this study, the CASIA-Iris-Distance database includes a large face area that includes both eyes in the input images as shown in Figure 3. As such, false detection cases, in which areas outside of the eye are improperly detected, can occur during eye detection that uses the described sub-block-based template matching alone. To resolve this problem, the present study uses the Adaboost eye detector in a search region that includes both eyes in the input image, as shown in Figure 3. The pupil and ocular ROIs are found within this region through sub-block-based template matching.

### 4.3. Deep ResNet-Based Ocular Recognition.

**Figure 4:**
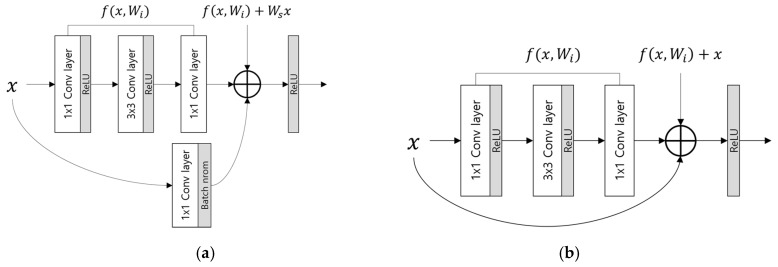
Examples of processes which involve short-cuts and identity mapping. (**a**) A process in which a 1 × 1 convolution (Conv) is used and the image size is adjusted to adjust the feature map channel when first starting to change the feature map size when processing each residual block. (**b**) A process that uses identity mapping when repeating the other residual blocks. In (**a**) and (**b**), rectified linear unit (ReLU) and batch normalization layer (batch norm) are included.

The existing CNN architectures that have been studied have demonstrated very good performance in the field of image processing and recognition. Various CNN models have been introduced, including AlexNet by Krizhevsky et al. [42], which comprises five convolutional layers (CL) and three fully connected layers (FCL), and Visual Geometry Group (VGG)-Net by Simonyan et al. [43], which comprises 16 or 19 CLs and three FCLs. Through these studies, it was discovered that performance improves as the layer depth increases. The architecture used in this study, deep ResNet, typically has 50, 101, or 152 layers [44]. If the depth of the layers increases without any supplementation, the training accuracy decreases when a learning is performed, and the model cannot converge on the global minimum. This is because features are lost as the model continues to go through processing and the existing image features are reduced through continued calculations. A previous study [44] resolves this problem using the concepts of short-cut (skip-connection) based residual blocks and identity mapping, as shown in Figure 4.

As seen in Figure 4, elemental-wise addition is performed via the short-cut to identity-map input x to the output where input x is processed through the number of convolution layers *f*(*x*). By doing so, the identities of the features that are continually calculated can be preserved even as many layers accumulate. The convolution layers calculated at the same time as this single skip-connection are bound together and called a single residual block. ResNet is based on these residual blocks, and each convolution layer is created from several residual blocks [44]. Table 2 shows a detailed description of the model used in this study for ResNet. In Table 2, Input shows the input layer; Conv shows the convolutional layer; and Max-pool shows the max pooling layer.

**Figure 5:**
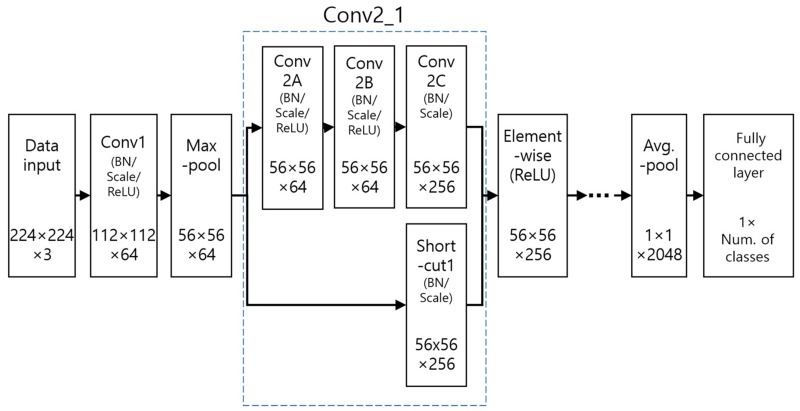
Detail description of residual block.

As shown in Table 2, each convolution layer comprises an accumulation of residual blocks. Each residual block comprises convolution layers with three small filter sizes of 1 × 1, 3 × 3, and 1 × 1, and this is called a bottleneck design. It performs fewer calculations than processing two 3 × 3 convolutions, and the performance is similar or better [44]. To reduce the output feature map size to half when each convolution layer starts as the residual blocks accumulate, the stride of the block’s first 1 × 1 convolution layer is set to 2 to reduce the feature map size, and the strides of the other blocks are set to 1 to maintain the feature map size as it is. Likewise, in the case of the shortcut, a 1 × 1 conv + BN is performed in the first shortcut to match the number of channels in order to reduce the feature size of each convolution layer. In the other shortcuts, the 1 × 1 conv is not performed and identity mapping which preserves the features is performed through an elemental-wise summation of the output of the previous block as it is. Figure 5 shows the residual blocks in more detail. Conv2_1 refers to the first block of Conv2 in Table 2. The three items in this block, Conv 2A, 2B, and 2C, refer to the 1 × 1, 3 × 3, and 1 × 1 filter sizes and convolutions, respectively. The scale in Figure 5 shows the magnification operation of the feature map value.

To train with the dataset used in this study, fine-tuning was performed based on the pretrained ResNet model. This is because hundreds of datasets are needed to train the weights of the many layers shown in Table 2, and the test dataset used in this study is inadequate for this. In this study, the pretrained ResNet model of He et al. [44] was used. This model was pre-trained using the ImageNet database, and this database comprises millions of images [45]. The model was used in the ImageNet large scale visual recognition competition (ILSVRC). Because of this, the size of the images was resized to the ImageNet data input size during the input data resizing process in this study. For fine-tuning at this time, the layers that were to be re-trained were selected. In this study, only the fully connected layer part of Conv5 in Table 2 was fine-tuned.

To train a CNN, several hyper-parameters and optimizers must be selected. Most training processes can be divided into forward processes and backward processes. A forward process initializes a given weight and uses this to perform calculations according to the model’s stages in order. Afterward, the ground-truth value that was originally attempted to obtain in the backward process and the results calculated by the forward process are compared to calculate the loss. This error is used and the weights are adjusted going backward to perform training. In the forward process, the activation function is considered important. Previously, a sigmoid function was normally used, but this required a lot of time and computation to calculate, so now a ReLU [46] function is usually used as the activation function. This ReLU function is often used because it is easy to calculate and does not produce negative values, and it shows somewhat better performance in making the training converge. Moreover, it does not require considerable computation to calculate the slope value for training [42,43,44]. The output obtained after the forward process is performed is compared with the ground-truth value, and a backpropagation process is performed to modify the weights using the stochastic gradient descent (SGD) method to optimize the weights of the training model. The first item which must be calculated at this time is the loss between the current results and the ground truth. How accurately this can be calculated determines whether the process will converge so that the training is completed properly. For the method used here, calculations were performed through basic multinomial logistic loss, and the output predicted through softmax [47] was used to perform the calculation. If the calculations are performed in this manner, it is possible to maintain more numerical stability when calculating the slope. Each of the results calculated by softmax function is used as an input to calculate the multinomial logistic loss [48].

### 4.4. Extracting Feature Vector and Calculating Matching Distance

In normal biometric studies, recognition performance is measured under two settings: closed world and open world. In the former, the classes of data are the same during training and testing. In the latter, the classes of data are different during training and testing. Under normal scenarios in biometrics, the classes of data may or may not be the same during training and testing. Therefore, an open world setting is more suitable for real-world applications. This study evaluated recognition performance in an open world setting. During this type of classification for biometrics, the output of the CNN’s fully connected layer is used, or the feature vectors extracted from the layer before the last fully connected layer are used to perform matching based on the matching distance with the feature vectors of the enrolled images. In a closed world setting, the classes of the data are the same during training and testing. Therefore, the output of the CNN’s fully connected layer can be used as-is. However, in an open world setting, the classes of data are different during training and testing. Therefore, feature vectors extracted from the layer before the last fully connected layer are used to perform recognition based on the matching distance with the feature vectors of the enrolled images. This study obtained the matching distance using Euclidean distance based on 2048 feature vectors extracted from Table 2’s average pooling layer. If the enrolled and input images are in the same class (genuine matching), a small Euclidean distance is calculated for them. Conversely, if they are in different classes (imposter matching), a large Euclidean distance is calculated for them. In this study, genuine matching occurred when this Euclidean distance was smaller than the threshold, and imposter matching occurred when it was larger than the threshold. Based on the training data, the distance at which the false acceptance error (FAR) and the false rejection error (FRR) were the same was set as the optimal threshold. FAR is the error of incorrectly accepting imposter data as genuine data, whereas FRR is that of incorrectly rejecting genuine data as imposter data. In general, FAR and FRR share a trade-off relationship, and the error in case FAR is similar to FRR is called as the equal error rate (EER).

## 5. Experimental Results with Analysis

### 5.1. Datasets and Data Augmentation

To evaluate the performance of the ocular recognition method proposed in this study, three types of open databases captured in an NIR camera environment were used to perform tests: CASIA-Iris-Distance, CASIA-Iris-Lamp, and CASIA-Iris-Thousand databases [49]. The distances between camera and user’s eye in case of collecting CASIA-Iris-Lamp and CASIA-Iris-Thousand databases are also in rear range, which are similar to that of CASIA-Iris-Interval. Therefore, the influence by NIR sources on the average illumination level of the center block was already tested in our experiments. Each of the databases was divided into two subsets, and two-fold cross validation was performed. For example, the CASIA-Iris-Distance database’s 282 classes, which include both eyes of the 141 people, were divided into sub-database 1 (DB1) and sub-database 2 (DB2) with 71 (142 classes) and 70 people (140 classes), respectively. 

Data augmentation was performed, and then training was conducted. The data augmentation is to increase the number of training data. The augmented data were used only for training, and original data were used for testing. In this way, training and testing were performed separately for two-fold cross validation. By doing so, the study aimed to prevent the problem of insufficient training data and overfitting in which the CNN trains excessively for the training data and the performance for the testing data is reduced. The mean accuracy obtained from two rounds of testing based on two-fold cross validation was used as the ultimate accuracy of the method proposed in this study. Table 3 below contains detailed descriptions of the experimental databases used in this study.

**Figure 6:**
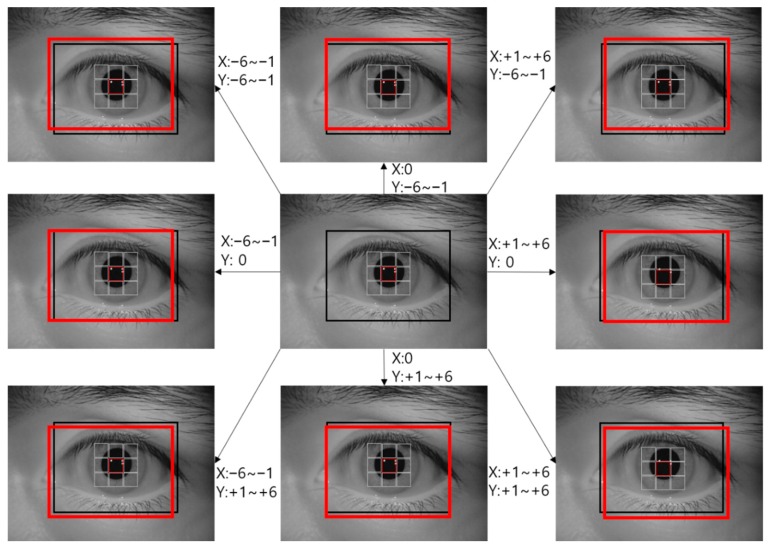
Example of data augmentation process used in this study.

For the training data, data augmentation was performed on the ROI areas found through the sub-block based template matching described in Section 4.2. Six pixels of translation and cropping in the up, down, left, and right directions were performed as shown in Figure 6 to augment the data by a factor of 169. Such translation and cropping-based data augmentation has been widely used in previous studies [42]. Through this, the misalignment between the enrolled and recognition images can be covered through CNN training, and the problem of inadequate training due to a small dataset can be resolved.

### 5.2. Training of CNN Model

**Figure 7:**
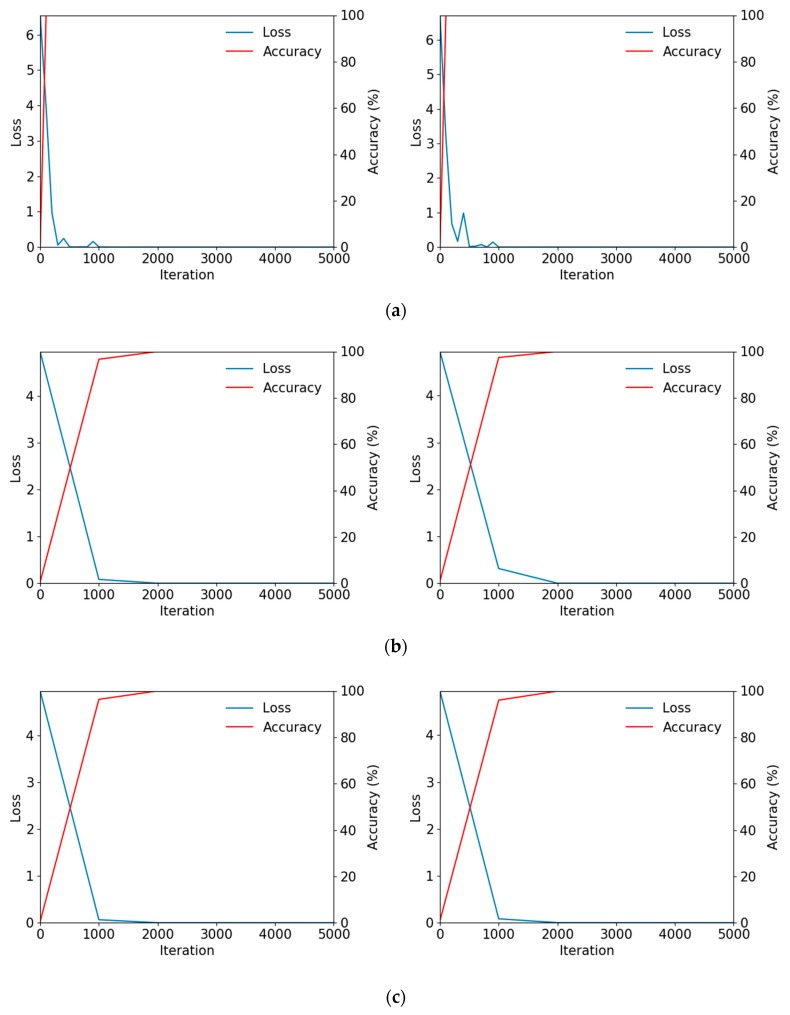
Examples of training loss and accuracy graphs with CASIA-Iris-Distance database. Left and right figures are the graphs of DB1 and DB2 training, respectively. Graphs of training by (**a**) ResNet-50, (**b**) ResNet-101 and (**c**) ResNet-152 models, respectively.

In the tests, the ResNet-50, 101, and 152 models were used to perform fine-tuning via the described augmented training data. During training, a stochastic gradient descent (SGD) optimizer [50] was used. Here, optimization was performed using a step policy as the learning rate policy, in which the gamma value was multiplied every fixed iteration. As one of SGD’s features, training was performed in the unit of mini-batch sizes. The number of iterations was calculated as the “number of training data/mini-batch size,” and the number of iterations here was defined as 1 epoch. In this study, training was performed for 3–10 epochs for each model. The learning rate was 0.0001. A small learning rate was used because fine-tuning was being performed using existing learned weights; the momentum value was 0.9, weight decay was 0.0001, and the gamma value was 0.1. Because the number of images in each dataset varied, the number of steps varied to match this and find optimal performance. To calculate the training loss, the multinomial logistic loss was calculated using the softmax function. Figure 7 below shows graphs of the training loss and training accuracy obtained during the training of ResNet-50, 101, and 152. As shown in Figure 7, as the training iterations increase, the training loss converges near 0 and the training accuracy converges near 100%. This shows that the training of the CNN model used in this study was performed successfully.

Training and testing for the method proposed in this study were performed on a desktop computer equipped with an Intel i7-975 3.33 GHz, 16 GB of RAM, and an NVIDIA GTX1070 graphic processing unit (GPU) [51]. The compute unified device architecture (CUDA) (version 8.0) and CUDA deep neural network library (cuDNN) (version 5.0) environments were used. Algorithms were implemented in OpenCV (version 3.3.0), Visual Studio 2015, and Windows Caffe (version 1.0.0) [52].

### 5.3. Testing of Proposed CNN-Based Ocular Recognition

For the first test, the recognition accuracy of a variety of ResNet models was measured for each of the test databases. As described in Section 5.1, the first-fold validation (DB1), second-fold validation (DB2), and mean accuracy were found to be in accordance with the two-fold cross validation. The recognition accuracy was measured via the EER described in Section 4.4. There are examples of existing studies in which the accuracy is assessed as the d′ (d prime) value [53]. The d′ value is a concept wherein the distance between the genuine and imposter distributions is calculated based on the mean and standard deviations of the two distributions. However, the d′ value is generally useful if the genuine and imposter distributions follow a Gaussian distribution, and it is difficult to use the value as an index for measuring accurate recognition performance if these distributions do not follow a Gaussian distribution. In this study, EER was used. As shown in Table 4, ResNet-101 showed the best recognition performance for the CASIA-Iris-Distance database, ResNet-50 for the CASIA-Iris-Lamp database, and ResNet-152 for the CASIA-Iris-Thousand database.

**Figure 8:**
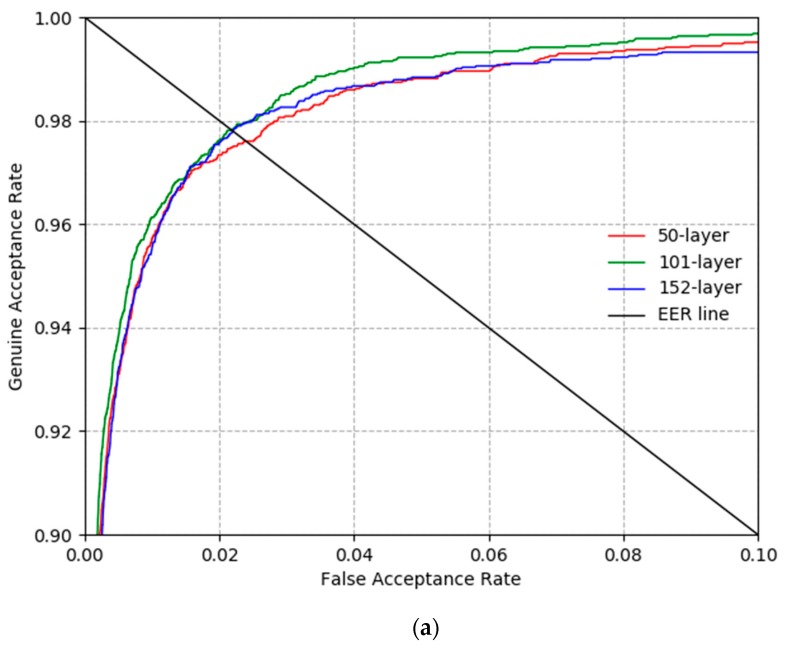

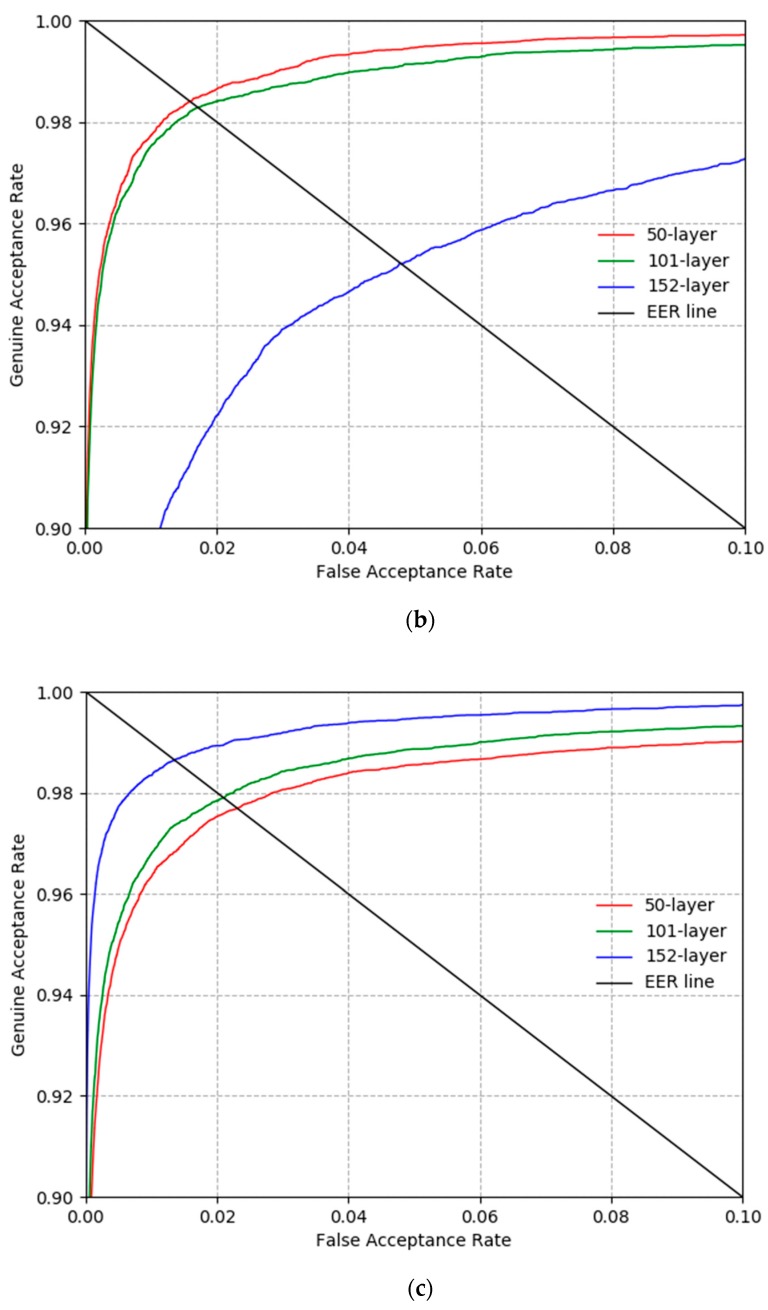
ROC curves of recognition by ResNet-50, 101, and 152 with (**a**) CASIA-Iris-Distance, (**b**) CASIA-Iris-Lamp, and (**c**) CASIA-Iris-Thousand.

Figure 8 shows the recognition accuracy measured in Table 4 in more detail as a receiver operating characteristic (ROC) curve. The genuine acceptance rate is calculated as 1 −FRR. Each graph is the mean graph of the two graphs found in the two-fold validations. From the results in Figure 8, it is clear that ResNet-101 showed the best recognition performance for the CASIA-Iris-Distance database, ResNet-50 for the CASIA-Iris-Lamp database, and ResNet-152 for the CASIA-Iris-Thousand database.

For the next test, the recognition performance was evaluated according to the size of the eye area ROIs obtained through the method described in Section 4.2. The test was performed on the CASI-Iris-Distance database because the size of the eye area that includes the iris in the CASIA-Iris-Distance database is smaller than that in the other two databases. Therefore, it is expected that the degree to which the area around the eye (periocular area) is included will change considerably, according to the changes in the size of the ROI; this will have a relatively large effect on the recognition performance. Based on the results in Table 4, ResNet-101 was used, which shows the highest recognition performance for the CASIA-Iris-Distance database. The average EER of the two-fold cross validation-based testing was found. As shown in Table 5, the recognition EER was the lowest when an ROI of 380 × 280 (width × height) pixels was used. The EER does not change significantly according to the change in the size of ROI in contrast to the recognition EER. From this, it can be known that the method used in this study was not greatly affected by changes in the size of the ROI. When the ROI was 380 × 400 (width × height) pixels, the EER increased further, and this is attributed to the ROI being larger vertically than horizontally, and it including more of the skin area in the vertical direction rather than on both edges of the eye, which are important for recognition. Based on these results, it was found that the EER can be reduced by making the ROI larger horizontally than vertically during recognition. The ROI of 300 × 260 shows the high EER, which means that too small ROI cannot include the important features of ocular region and consequent recognition error increases.

Based on [54], we compared the recognition accuracies with pre-trained ResNet without fine-tuning and fine-tuned ResNet on CASIA-Iris-Distance database. The pre-trained ResNet was pre-trained using the ImageNet database, and this database comprises millions of images as explained in Section 4.3 [45]. As shown in Table 6, our fine-tuned ResNet shows the higher accuracy than pre-trained ResNet without fine-tuning.

**Figure 9:**
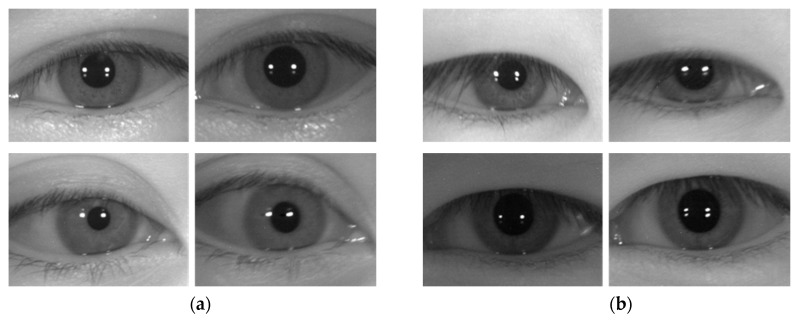

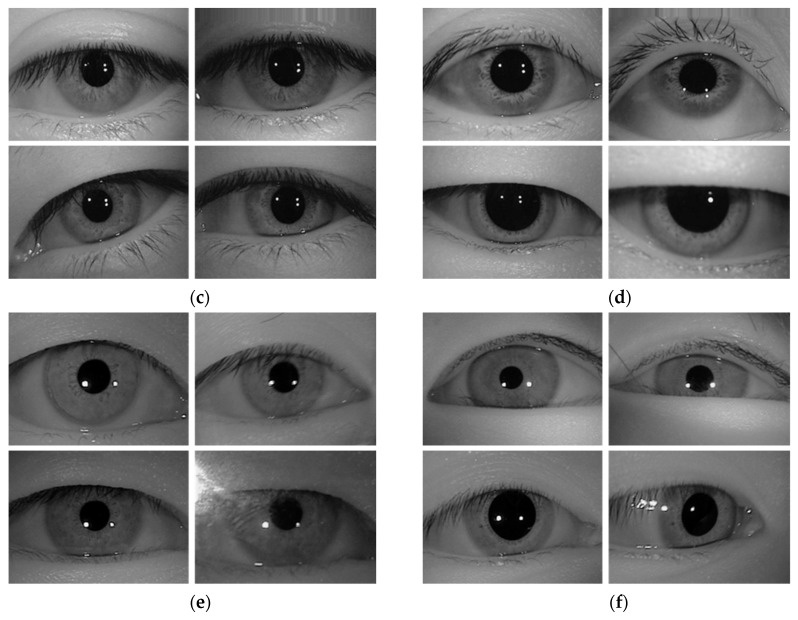
Examples of correction recognition cases achieved through the method proposed in this study. (**a**,**b**) CASIA-Iris-Distance database. (**c**,**d**) CASIA-Iris-Lamp database. (**e**,**f**) CASIA-Iris-Thousand database. In (**a**–**f**), the image on the left is the enrolled image, and the image on the right is the recognition attempt image.

Figure 9 below shows examples of correct recognition cases achieved through the method proposed in this study. As shown in Figure 9, correct recognition occurred even in cases where there were some differences between the enrolled image and the recognition image including the level of eye openness, in-plane rotation, off-angle, scaling, and specular reflection on glasses surface.

**Figure 10:**
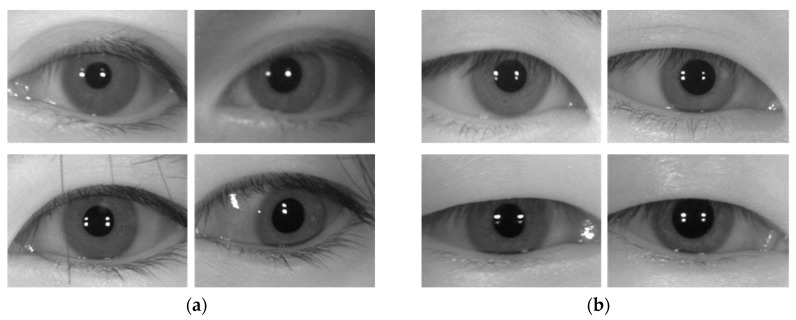

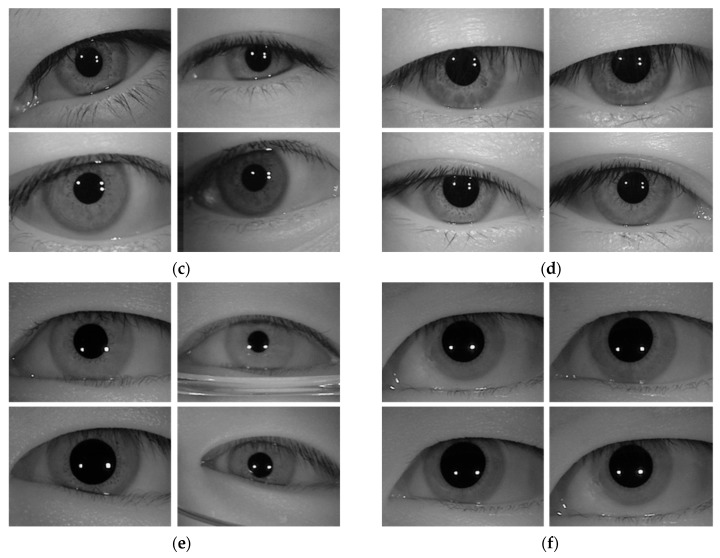
Examples of recognition error cases due to the method proposed in this study. (**a**,**b**) CASIA-Iris-Distance database. (**c**,**d**) CASIA-Iris-Lamp database. (**e**,**f**) CASIA-Iris-Thousand database. (**a**,**c**,**e**) are FR cases. (**b**,**d**,**f**) are FA cases. In (**a**–**f**), the image on the left is the enrolled image, and the image on the right is the recognition attempt image.

Figure 10 below shows false rejection (FR) and false acceptance (FA) cases that occurred due to the method proposed in this study. As shown in Figure 10a,c,e, FR cases occur when severe in-plane rotation and off-angle happen, or glasses frame is included in the image. As shown in Figure 10b,d,f, FA cases happen when the enrolled and recognition images are similar.

### 5.4. Comparisons with Proposed and Existing Methods

In the next test, the recognition accuracy of the method proposed in this study and existing methods were compared for the CASIA-Iris-Distance, CASIA-Iris-Lamp, and CASIA-Iris-Thousand databases. In Table 7, Table 8 and Table 9, the method proposed in this study showed higher recognition accuracy than the existing research methods. 

The recognition method that used the deep features extracted from ResNet and the simple ocular area detection method employed by this study showed higher recognition accuracy than the existing handcrafted feature-based method or the traditional machine learning-based method.

As the next experiment, we check whether the recognition performance is related to the part of iris in the ocular image. In previous research [63], they compared the accuracies only by periocular region, iris region, and fusion, respectively. However, the periocular region included both iris and periocular regions in their research, and the accurate effect only by periocular region without iris area was not measured. Therefore, we used the following scheme. With CASIA-Iris-Distance database, the segmented iris regions were painted as black pixels. Then, training and testing with our deep CNN using these images were performed. As shown in Table 10, the recognition error with these images (using periocular region without iris area) is higher than that by proposed method (using whole ocular region). Based on this result, we can find that the recognition performance is related to the part of iris in the ocular image and the iris region is also necessary for ocular recognition of high accuracy. In addition, as shown in Table 10, the accuracy by using iris area without periocular region was measured, and it is lower than that by using whole ocular region. Based on these results, the ocular region including both periocular and iris areas is necessary for high recognition accuracy. 

As the next experiment, we performed the experimental comparisons based on the [63]. In this paper, they considered that the periocular region includes both iris and periocular regions. Therefore, their method based on the periocular region corresponds to our method using the whole ocular region. As shown in Table 11, our proposed method shows the higher accuracy than those by using iris area without periocular region and the fusion of iris and periocular regions.

In this research, we also performed the experimental comparisons based on the [64]. As shown in Table 12, the method with our augmented database shows the higher accuracy than that with the augmented database based on affine transform [64].

As the next test, in order to include the more variations in augmented data, we performed the additional experiments to compare the accuracy by using our augmented data (explained in Section 5.1) with that by using CASIA-Iris-Thousand database [49] for training our CNN model. The reason why we used CASIA-Iris-Thousand database is that the number of images and classes are the largest among all the CASIA iris databases including CASIA-Iris-Syn database [49]. As shown in Table 13, we can find that the case of training with our augmented database shows the higher accuracy than that by training with CASIA-Iris-Thousand database.

For the final test, a processing speed comparison was performed on the sub-block based template matching ocular ROI detection method used in this study, a two-circular edge detector [6], and a CNN-based iris segmentation method [65]. The test environment was described in Section 5.2. As shown in Table 14, it can be seen that the sub-block-based template matching ROI detection method used in this study requires less processing time than the existing two-circular edge detector [6] and the CNN-based iris segmentation method [65]. Table 14 also shows the time for performing ocular recognition with the deep ResNet proposed in this study based on the specified ROIs, and the ultimate processing time was found to be 115 ms. From this, it can be known that the recognition system proposed in this study can operate at a speed of about 5.3 (=1000/(73 + 115)) frames per second.

### 5.5. Analysis of Feature Maps Extracted from CNN Convolutional Layers and Discussion

**Figure 11:**
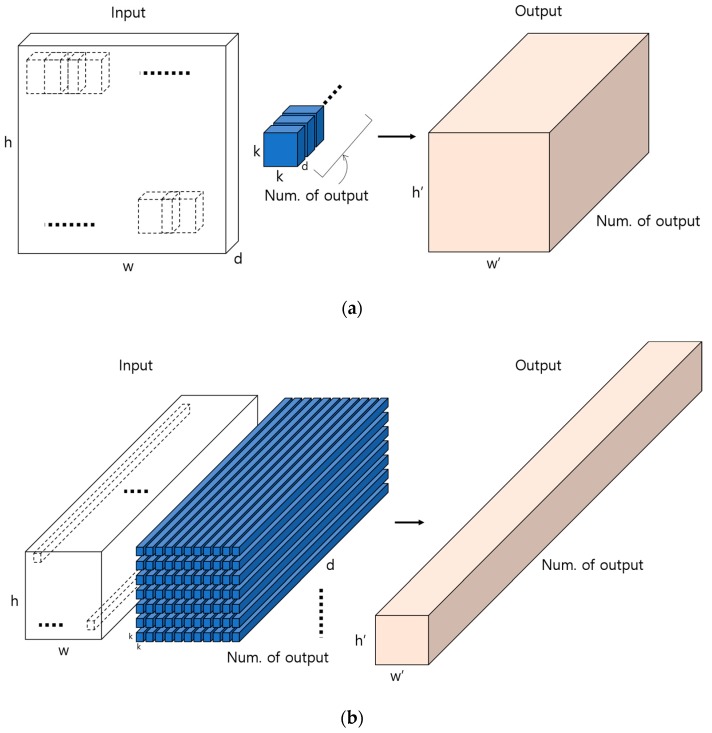
Example of obtaining output feature map via the convolutional layer. (**a**) Example of creating output feature map (w′ × h′ × Num. of output)) using as many as k × k filters of the same depth as the number of output on the input image (w × h × d). (**b**) Example of convolution calculations and finding the output feature map when the input feature map’s depth increases afterward.

In general, a k × k × d size filter is used on a w × h × d size input, as shown in Figure 11a, via a calculation in the convolution layer to find an output feature map of the size w′ × h′ × Num. of output. Here, the number of filters used is the number of output (Num. of output) such that the depth of the ultimate output feature map is the number of output. Figure 11b shows an example of convolution calculations and finding the output feature map when the input feature map’s depth increases.

**Figure 12:**
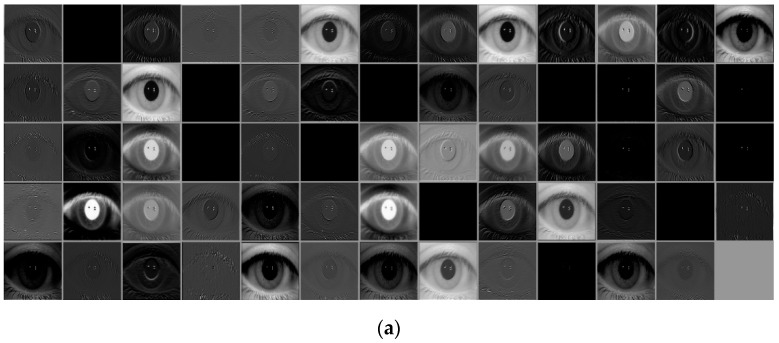

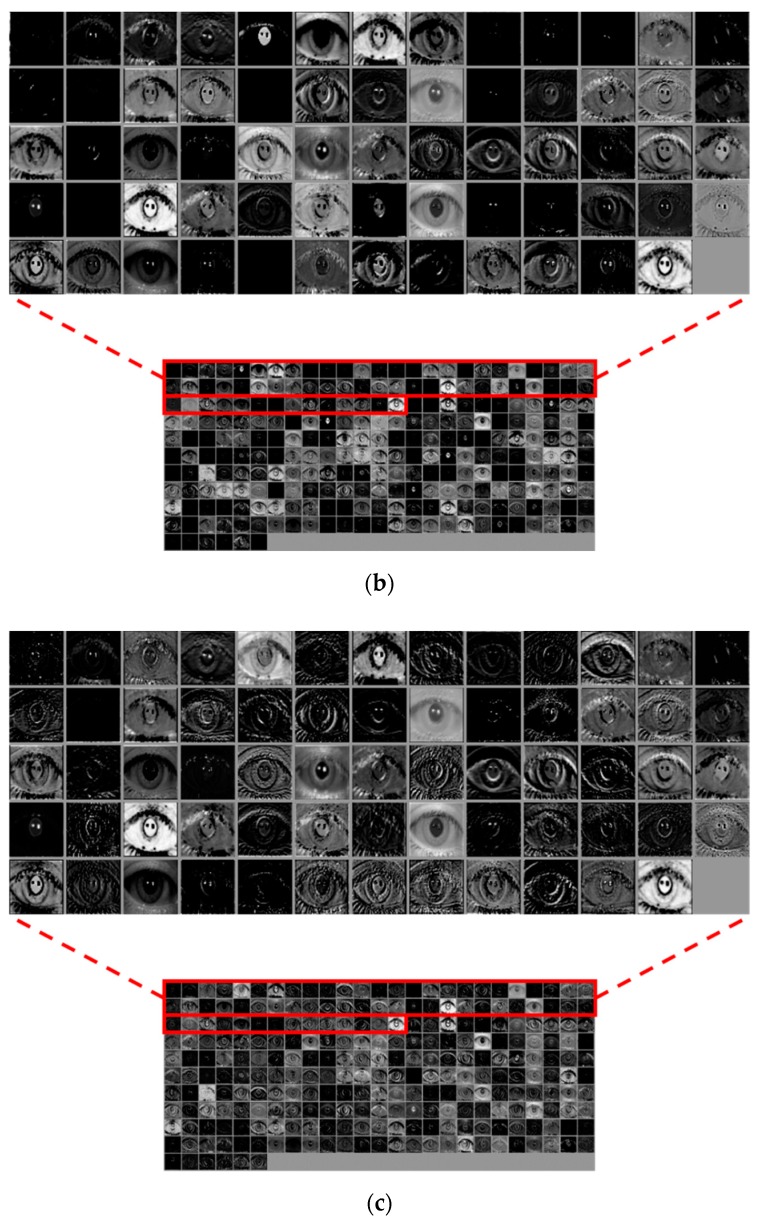

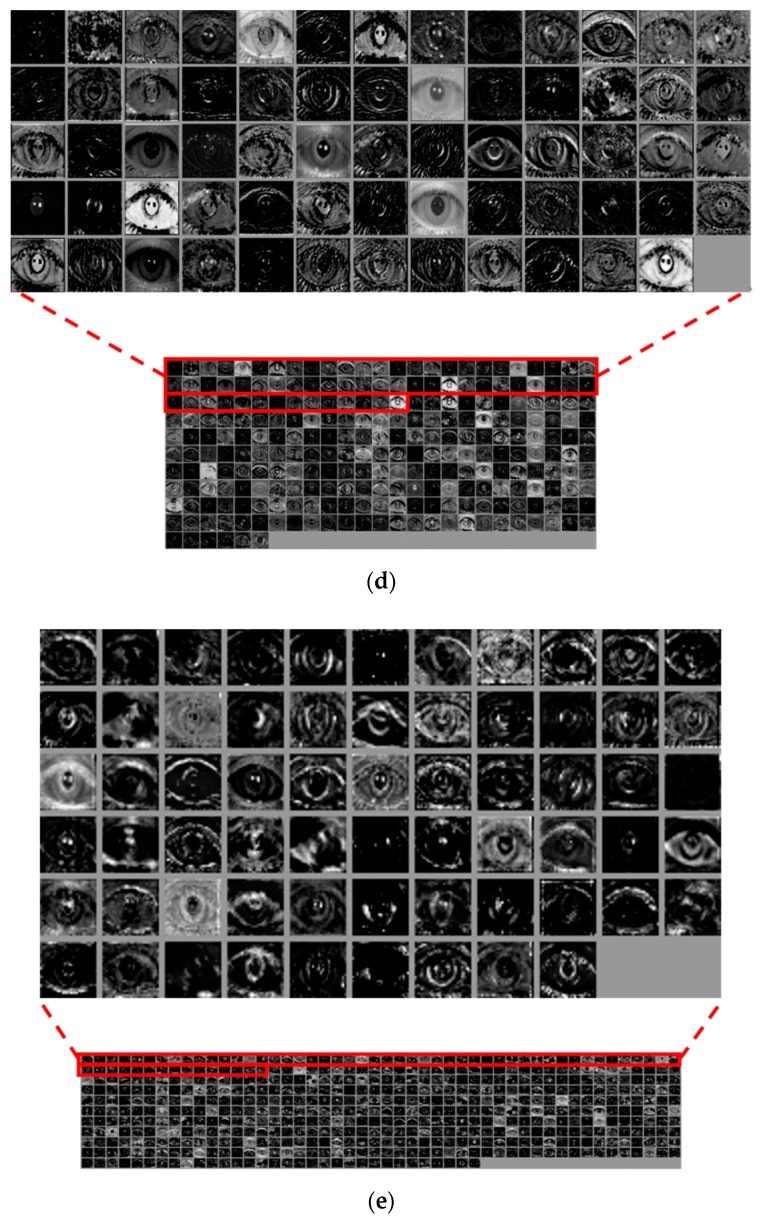

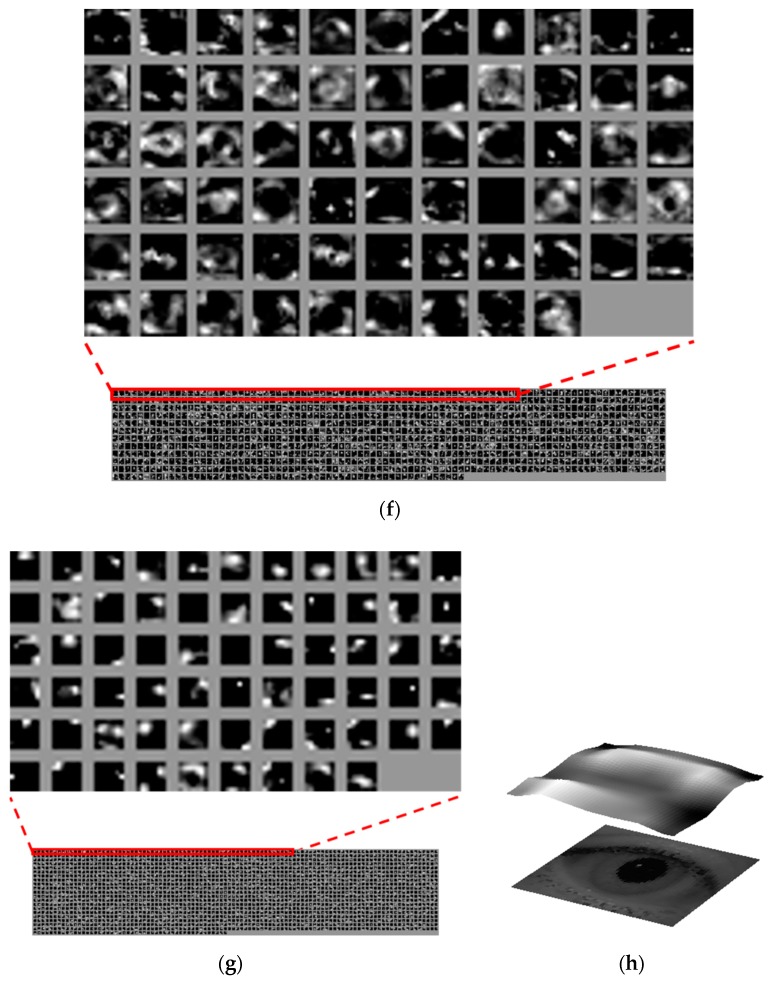
Examples of features maps extracted from each layer for the input ocular images. Feature maps from (**a**) Conv1 of Table 2, (**b**–**d**) the first, second, and third iterations of residual blocks in Conv2 of Table 2, respectively, and (**e**–**g**) the last residual blocks in Conv3, Conv4, and Conv5 of Table 2, respectively. (**h**) 3-dimensional feature map image that is obtained by averaging all the feature map values of (**g**).

Based on Figure 11, this section analyzes the feature maps extracted from each layer of deep ResNet for the input ocular images as shown in Figure 12. As described in Figure 11, the output feature map’s depth increased as the layers became deeper. Because this is difficult to express in 3D form, the feature maps for each depth are shown in the order from the top left to the bottom right, as seen in Figure 12. Figure 12a shows the feature map from Conv1 of Table 2. Figure 12b–d show the feature maps from the first, second, and third iterations of residual blocks in Conv2 of Table 2, respectively. Further, Figure 12e–g show the feature maps from the last residual blocks in Conv3, Conv4, and Conv5 of Table 2, respectively. For example, the feature maps obtained from Table 2’s Conv1 have a size of 112 × 112 × 64; therefore, 64 feature maps of size 112 × 112 are shown in the order from the top left to the bottom right, in Figure 12a. 

As shown in Figure 12, feature maps are extracted from deeper convolutional layers, and as more abstract features are extracted, the area of the dominant feature in a feature map tends to grow larger. For example, in Figure 12a, features exist that show the original ocular shape and high-frequency edge components; however, in Figure 12g, the original ocular shape has disappeared, and the feature map consists of abstracted low-frequency features. Further, in Figure 12b–d, more convolution layers are performed than that in Figure 12a, and the ocular shape in the feature map disappears further. However, owing to the structure that preserves the original feature map before the convolution operation passes through residual blocks, the original ocular shape does not disappear entirely, and it can be seen that it is preserved to some degree. In Figure 12f,g, it can be seen that convolution layers have become even deeper, and the original ocular shape is almost not preserved. Further, it can be seen that the characteristics of low-frequency features, which are large and abstract in the feature map of Figure 12g, compensate to some degree for the reduction in recognition performance caused by misalignment between the enrolled and recognition images owing to the rough detection of the ocular area proposed in this study. Figure 12h shows 3-dimensional feature map image that is obtained by averaging all the feature map values of Figure 12g in the channel (depth) direction, as well as the original ocular images. As shown in this figure, the magnitudes of feature map values are also large in the rough periocular region, which can prove that important features can be extracted from whole ocular region instead of only iris area.

Most of conventional studies on ocular recognition requires the procedure of iris segmentation [8,13,14,16,17,22,23,24,25,26,27,35], as shown in Table 1. This procedure includes the accurate detection of the boundaries and centers of pupil and iris, which takes much processing time and its accuracies are affected by the image quality and noises. Although there are previous studies without iris segmentation [18,20,21] as shown in Table 1, the recognition accuracies of their methods are low. Therefore, we propose ocular recognition based on rough pupil detection which does not include the procedure of accurate iris segmentation.

In our method, the rough pupil detection is performed by sub-block-based template matching as shown in Figure 2 and Figure 3. As shown in these figures, the boundaries and centers of pupil and iris are not detected. Instead, the rough box position including pupil is located. Experimental results show that the positional difference between pupil centers in enrolled and recognized images by our sub-block-based template matching is about 7~10 pixels in X and Y axes, which are much larger than that by conventional iris segmentation method (in most cases, the difference is less than 1~2 pixels). However, the processing speed by our sub-block-based template matching is much faster than that by conventional iris segmentation [6,65] as shown in Table 14. In addition, deep learning-based method is used in our research, and it could resolve the problem of reduced recognition accuracies due to the large difference between pupil centers in enrolled and recognized images by our sub-block-based template matching. Our deep learning-based method could also compensate the intra-class variation caused by openness of eye, in-plane rotation, off-angle, and specular reflections on glasses surface as shown in Figure 9.

Although the further extension of area, e.g., by the nose can produce the better accuracy, this kind of method cannot be used for the case that the nose area is occluded by wearing mask and its performance can be affected by the existence of mustache. 

The accuracy by iris recognition can be better than that by our ocular recognition in case that the quality and image resolution of iris image are good. However, in case that the quality and image resolution are not good, the accuracy by our method using both iris and periocular regions is better than that only by iris or periocular recognition. This was proved as shown in Table 10. Our method can be difficult to be used in safety critical areas which requires high recognition accuracy. However, we can expect that our ocular recognition can be used for more reliable identification of people than iris or face recognition in case that the quality and image resolution of iris image are not good when the image is captured by the moving and uncooperative people at a distance with image blurring or other facial components including nose or mouth are not visible by wearing masks.

## 6. Conclusions

This study proposed the NIR camera-based ocular recognition method that uses deep features without any iris segmentation. The method uses a roughly identified eye area for recognition, without going through a process for detecting the pupil and iris boundary, the eyelid, and eyelashes, as this process requires a long processing time and its performance is affected by the image quality. To prevent decrease in recognition performance due to the roughly identified eye area, an ocular area somewhat larger than the iris area is used to perform recognition. Further, the method uses deep ResNet to resolve the problem of misalignment between the enrolled and recognition images, which can occur because of the recognition based on a roughly detected eye area, as well as the problem of reduced recognition performance caused by this misalignment. The proposed method analyzes the feature maps from various convolutional layers of the deep ResNet in order to examine the features that are important for ocular recognition in each of the layers. The results of tests using three open databases showed that the method proposed in this study has higher recognition accuracy than that of existing methods. However, it was found that FR cases occurred when severe in-plane rotation and off-angle happen, or glasses frame is included in the image. In addition, FA cases happened when the enrolled and recognition images were similar.

To resolve these problem, we plan to conduct a study in the future on a multimodal biometrics method that improves recognition performance by combining recognition information from both eyes. Further, we plan to study a method that can enlarge the CNN model’s receptive field and extract features in order to resolve the problem of reduced recognition performance caused by the misalignment between the enrolled and the recognition images owing to the rough detection of the ocular region. In addition, we would plan to collect twin’s iris dataset and have experiments with this self-collected dataset as future work.

## Figures and Tables

**Table 1:** Comparisons of proposed and previous researches (d′, EER, GAR, and FAR mean d-prime value, equal error rate, genuine acceptance rate, and false acceptance rate, respectively. Their concepts are explained in Section 4.4 and Section 5.3).

Iris Segmentation	Periocular Region	Using Feature	Method	Accuracy	Advantage	Disadvantage
Included	Not used	Hand-crafted feature	Log Gabor filter, LDA, and BSA [35]	EER of 3.12~3.465% (A)	Captured iris images are less affected by external light than those from a visible light camera	Equipment becomes large-sized and expensive due to use of NIR camera and NIR lighting
			Pre-classification and in-plane rotation angle estimation for both eyes, then matching via bit-shifting [8]	EER of 4.3006% (A)		
	Used		Fusion-OTSDF, correlation filtering, and MAP estimation [22]	EER of 23.83~26.81% (C)		
			Fusion and classification with RDF using PHOG and gist method [23]	GAR of 61.5% at 0.1% FAR (A) GAR of 21% at 1% FAR (C)		
			Adaboost and OSIRIS [24]	EER of 3.6~3.9% (A)		
		Deep feature	Using three CNNs [26]	EER of 3.04~3.08% (A) EER of 10.36% (B) EER of 16.25~17.9% (E)	By using visible light camera without NIR lighting, equipment is small-sized and low cost, has no reflected light due to NIR lighting	- Iris quality is slightly lower than a normal NIR environment image due to nearby lighting noise and ghost effects - Difficult to capture iris patterns in visible light for races with little melanin pigment
			Fusion of the recognitions by iris, ocular, and the area larger than ocular [27]	EER of 5.7% (E)		
Not included		Hand-crafted feature	GOH, PDM, m-SIFT, and fusion by weighted SUM rule [18]	EER of 18.8% (C)	Captured iris images are less affected by external light than those from a visible light camera	Equipment becomes large-sized and expensive due to use of NIR camera and NIR lighting
			Quadratic correlation filter [21]	Classification accuracy of 75% (H)		
			Conversion of periocular region into polar-coordinates and matching via LBP and adaptive bit shifting [20]	EER of 10.0172% (A)		
			Multi-order statistical descriptors [32]	Classification accuracy of 93.33% (M)	Intensive training is not required	Performance enhancement is limited by using hand-crafted feature
			Multi-frame super resolution reconstruction based on deblurring by CNN [33]	EER of 19.54~28.06% (N)	Image resolution and blurring of ocular image can be enhanced	Using hand-crafted feature and unsophisticated matcher produces low recognition accuracy
Included	Not used	Deep feature	Iris recognition with off-the-shelf CNN features [36]	Recognition accuracy of 98.8% (G) Recognition accuracy of 98.7% (H)	High recognition accuracy	- Performance is affected by the accurate iris segmentation - The complexity of DenseNet-201 is large, which requires huge training time.
			DeepIris based on pairwise filter bank [37]	EER of 0.15% (I) EER of 0.31% (J)	Measure the accuracies according to various capturing distances and types of camera sensors	- Performance is affected by the accurate iris segmentation - Performance enhancement is limited by using shallow CNN
			DeepIrisNet [38]	EER of 2.19% (K) EER of 1.82~2.4% (L)	Compare the accuracies according to various types of segmentation method, in-plane rotations, input size, train size, and network size.	- Performance is affected by the accurate iris segmentation - Performance enhancement is limited by using shallow CNN
Not included	Used		Deep ResNet-based recognition (Proposed method)	EER of 2.1625% (A) EER of 1.595% (F) EER of 1.331% (G)	Not require iris segmentation while maintaining the accuracy by deep ResNet	Require the procedure of intensive training of ResNet

**Table 2:** Detailed description of each layer in our deep ResNet (1(2*) means that a stride of 2 is used only when processing each first residual block where the feature map size is reduced to half; otherwise 1 is used) (2** means that it is applied only to a 1 × 1 convolution, and it is not applied to identity mapping) (during each iteration, 1 × 1 convolution + batch normalization (BN) is performed only during the first iteration, and identity mapping is performed during the other iterations) (Conv3–Conv5 include bottleneck structures).

Layer Name	Size of Feature Map (Height × Width × Channel)	Number of Filters	Size of Filter	Number of Padding	Number of Strides	Number Iteration of Residual Block
Input	224 × 224 × 3					
Conv1	112 × 112 × 64	64	7 × 7 × 3	3	2	1
Max-pool	56 × 56 × 64	1	3 × 3 × 1	0	2	1
Conv2	56 × 56 × 256	64	1× 1 × 64	0	1	3
		64	3 × 3 × 64	1		
		256	1 × 1 × 64	0		
(short-cut)	56 × 56 × 256	256	1 × 1 × 64	0	1	
Conv3	28 × 28 × 512	128	1 × 1 × 256	0	1(2*)	- 4 in case of ResNet-50 and ResNet-101 - 8 in case of ResNet-152
		128	3 × 3 × 128	1	1	
		512	1 × 1 × 128	0		
(short-cut)	28 × 28 × 512	512	1 × 1 × 256	0	2**	
Conv4	14 × 14 × 1024	256	1 × 1 × 512	0	1(2*)	- 6 in case of Resnet-50 - 23 in case of Resnet-101 - 36 in case of Resnet-152
		256	3 × 3 × 256	1	1	
		1024	1 × 1 × 256	0		
(short-cut)	14 × 14 × 1024	1024	1 × 1 × 512	0	2**	
Conv5	7 × 7 × 2048	512	1 × 1 × 1024	0	1(2*)	3
		512	3 × 3 × 512	1	1	
		2048	1 × 1 × 512	0		
(short-cut)	7 × 7 × 2048	2048	1 × 1 × 1024	0	2**	
Average pooling layer	1 × 1 × 2048	1	7 × 7 × 1	0	1	1
Fully connected layer (Softmax)	1 × number of classes					1

**Table 3:** Detail descriptions of experimental databases.

Category	Number of Classes		Number of Images			
			Before Augmentation		After Augmentation	
	DB1	DB2	DB1	DB2	DB1	DB2
CASIA-Iris-Distance	142	140	2080	2056	351,520	347,464
CASIA-Iris-Lamp	408	408	8054	8036	1,361,126	1,358,084
CASIA-Iris-Thousand	1000	1000	9946	9946	1,680,874	1,680,874

**Table 4:** Comparative EERs with each dataset by various ResNet architectures (unit: %).

ResNet Model	CASIA-Iris-Distance			CASIA-Iris-Lamp			CASIA-Iris-Thousand		
	DB1	DB2	Average	DB1	DB2	Average	DB1	DB2	Average
50-layer	2.576	1.971	2.2735	1.505	1.685	1.595	2.091	2.524	2.3075
101-layer	2.138	2.187	2.1625	1.702	1.738	1.72	1.431	2.570	2.0005
152-layer	2.103	2.264	2.1835	5.203	4.372	4.7875	1.588	1.074	1.331

**Table 5:** Comparisons in recognition accuracies according to ROI crop size (unit: %).

	500 × 400 Pixels	500 × 280 Pixels	380 × 400 Pixels	380 × 280 Pixels	300 × 260 Pixels
EER	2.184	2.282	3.334	2.163	2.955

**Table 6:** Comparative EERs with pre-trained ResNet without fine-tuning and fine-tuned ResNet (unit: %).

Method	DB1	DB2	Average
Pre-trained ResNet without fine-tuning	11.504	9.728	10.616
Fine-tuned ResNet (proposed method)	2.138	2.187	2.1625

**Table 7:** Comparative EERs on CASIA-Iris-Distance database (unit: %).

Method	EER
Cho et al. [20]	10.0172
Shekar et al. [55]	8.64
Zhao et al. [30]	4.9
Shin et al. [8]	4.3006
Oishi et al. [24]	3.6~3.9
Sharifi et al. [35]	3.12~3.465
Lee et al. [26]	3.04~3.08
Tan et al. [56]	2.9
Proposed method	2.1625

**Table 8:** Comparative EERs on CASIA-Iris-Lamp database (unit: %).

Method	EER
Uhl et al. [57]	12.9
Ribeiro et al. [29]	3.92
Abdullah et al. [58]	2.37
Proença et al. [59]	2.6
Nigam et al. [60]	2.13
Zhang et al. [61]	2.05
Li et al. [62]	2.02
Proposed method	1.595

**Table 9:** Comparative EERs on CASIA-Iris-Thousand database (unit: %).

Method	EER
Drozdowski et al. [31]	8.27
Proença et al. [59]	3
Li et al. [62]	2.59
Proposed method	1.331

**Table 10:** Comparative EERs on CASIA-Iris-Distance database in case of using whole ocular region or using periocular region without iris area (unit: %).

Method	EER
Using periocular region without iris area	5.2506
Using iris area without periocular region	3.8952
Using whole ocular region (proposed method)	2.1625

**Table 11:** Comparative EERs on CASIA-Iris-Distance database for measuring the effect of iris or periocular region based on [63] (unit: %).

Method	EER
Using iris area without periocular region	9.5069
Using periocular region (proposed method)	2.1625
The fusion of iris and periocular regions [63]	6.2207

**Table 12:** Comparative EERs on CASIA-Iris-Distance database in case of training with augmented database based on affine transform [64] with our augmented database (unit: %).

Method	EER
Training with augmented database based on affine transform [64]	4.2352
Training with our augmented database	2.1625

**Table 13:** Comparative EERs on CASIA-Iris-Distance database in case of training with CASIA-Iris-Thousand database or training with our augmented database (unit: %).

Method	EER
Training with CASIA-Iris-Thousand database	2.5471
Training with our augmented database	2.1625

**Table 14:** Comparisons of processing speed by proposed method with other methods (unit: ms).

	Proposed Method (Sub-Block Based Template Matching)	Two-Circular Edge Detector [6]	CNN-Based Iris Segmentation [65]	Ocular Recognition (Feature Extraction + Distance Matching)
Processing time per an image	73	986	210	115
